# Supplementary material for: Mathematical model linking telomeres to senescence in Saccharomyces cerevisiae reveals cell lineage versus population dynamics
Source: Nat Commun. 2025 Jan 25;16:1024. doi: 10.1038/s41467-025-56196-z (PMC11762778; doi:10.1038/s41467-025-56196-z)
Supplement: Supplementary file 1 — Supplementary Information [file 41467_2025_56196_MOESM1_ESM.pdf]

Supplementary Information

Mathematical model linking telomeres to senescence in *Saccharomyces cerevisiae* reveals cell lineage versus population dynamics

Anaïs Rat, Veronica Martinez Fernandez, Marie Doumic, Maria Teresa Teixeira and Zhou Xu

corresponding authors. Emails: Marie.Doumic@inria.fr, Teresa.teixeira@cnrs.fr

This pdf includes:

Supplementary Figures 1-6

Supplementary Methods

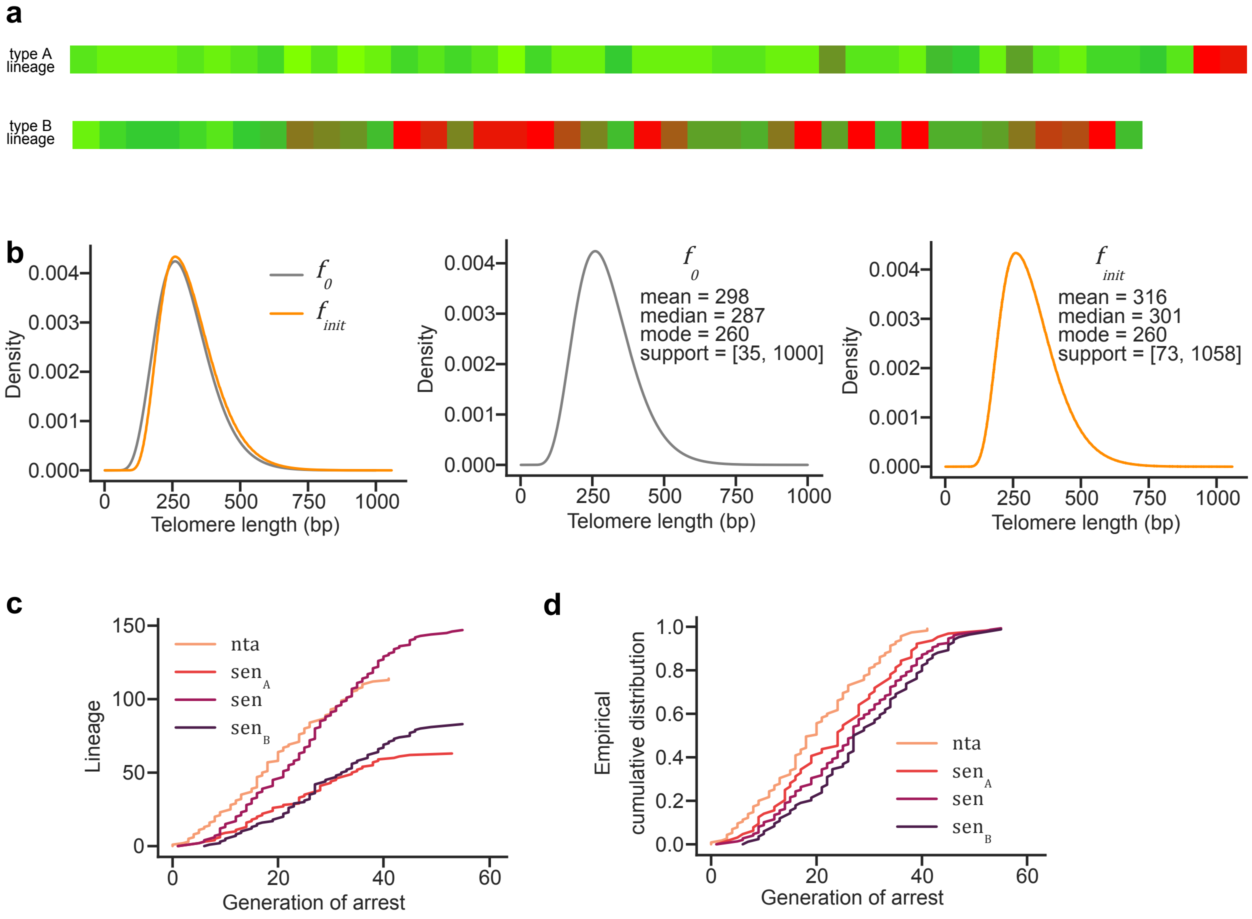

Supplementary Fig. 1: Model description details. ***a*** *Enlarged examples of type A and type B lineages taken from Fig. 1C.* ***b*** *Initial telomere lengths distributions.* f_0 *has been computed in* [1] *by adapting the approach of* [2] *to a yeast strain expressing telomerase RNA subunit under a conditional promoter as used in microfluidics experiments* [3,4]. f_{init} *corresponds to the slight modification of* f_0 *described in Methods with parameters of Table 1.* ***c*** *Experimental generation of arrest classified by lineage type and type of arrest (non-terminal or senescent arrest, as extracted from Fig. 1c. nta: generation of the first non-terminal arrest;* sen_A*,* sen_B*: generation of first senescent arrest for* type A, B *cells; sen: generation of first senescent arrest for all types.* ***d*** *same as in (c) normalized to obtain empirical cumulative distribution.*

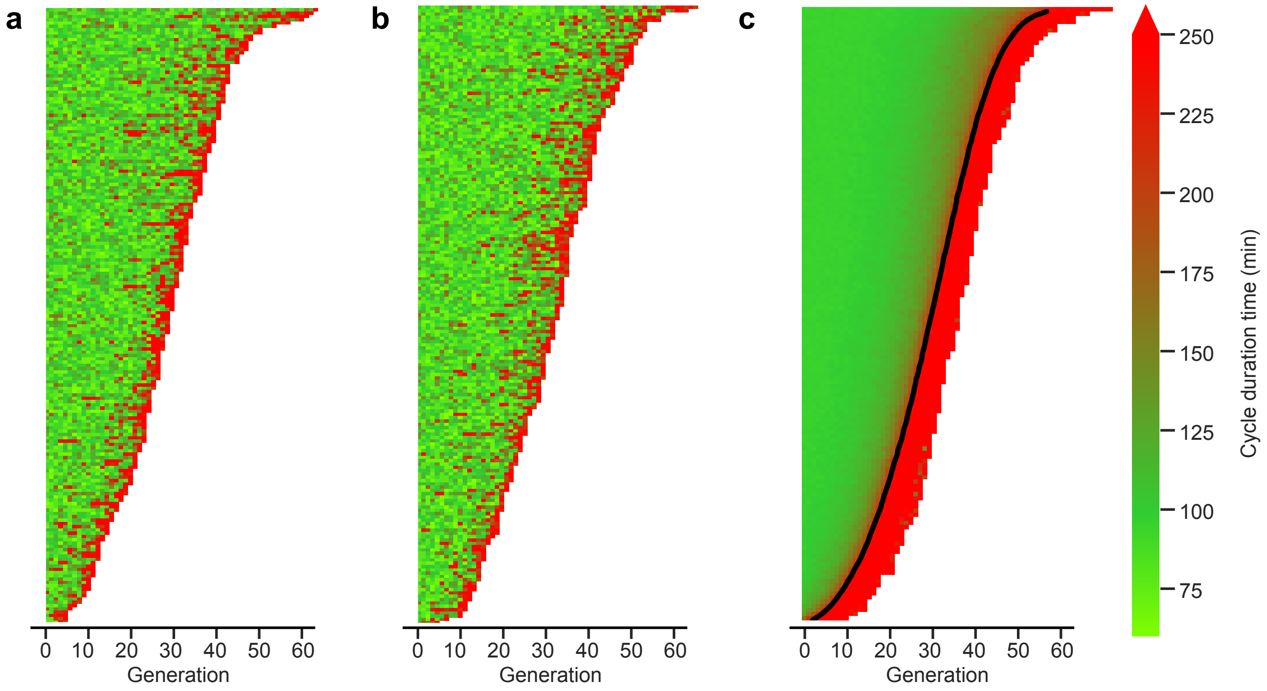

Supplementary Fig. 2: Simulations of single lineage experiments. a-b Simulated data of one microfluidics experiment with the best-fit model, as in Fig. 2C. c Average over 1000 random simulations of the microfluidics experiment using the best-fit model (parameters in Table 1).

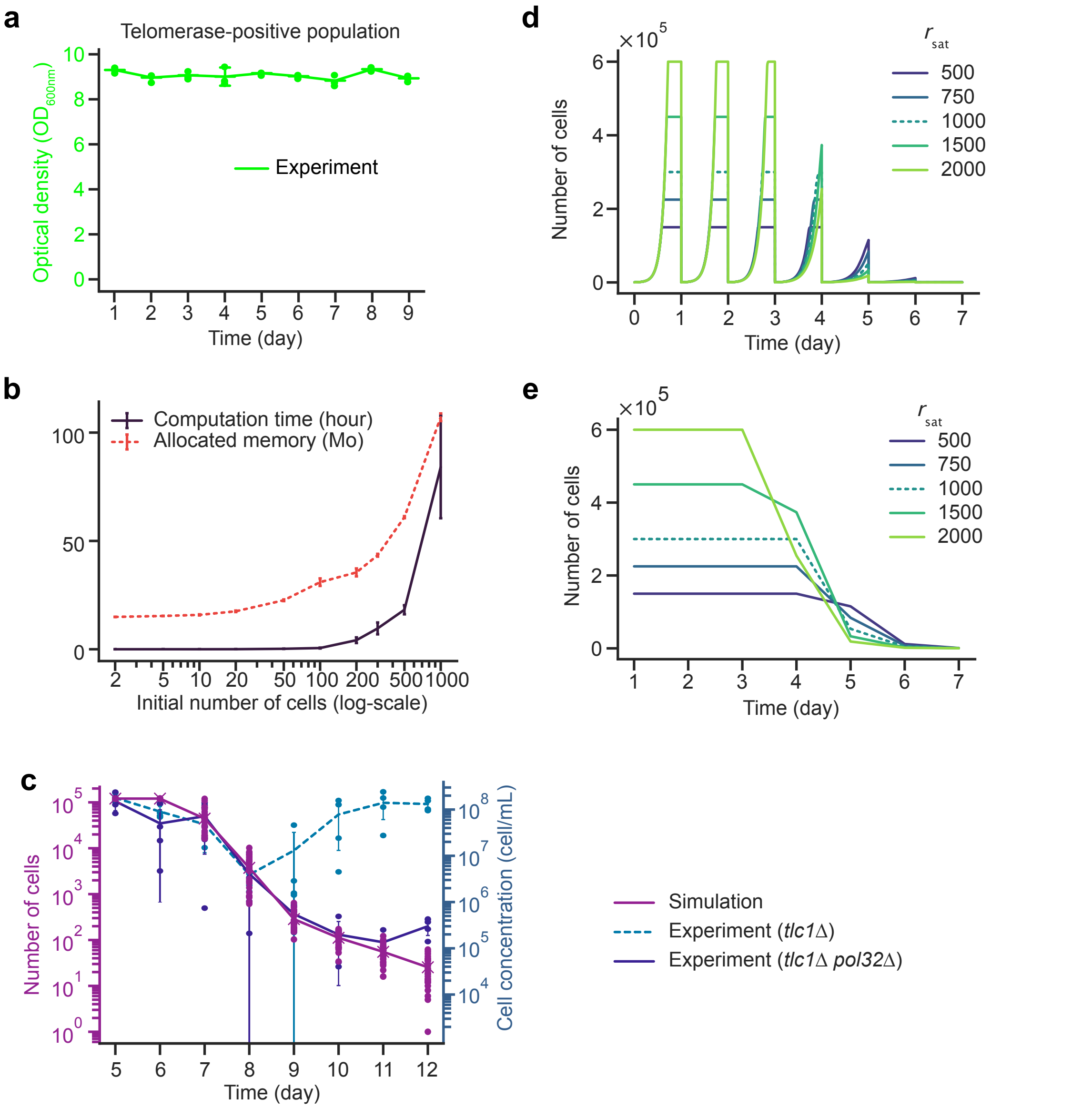

Supplementary Fig. ***3: Study to establish the parameters for the population simulation. a*** *Experimental data of cell growth as in Fig. 3B in telomerase-positive conditions to determine the ratio r_{sat} to generate Fig. 3B.* ***b*** *Computation time and allocated memory in MegaOctets (Mo) with respect to N_{init}.* ***(c)*** *Comparison between experimental data of telomerase-negative cells, mutated or not for* POL32 *taken from reference 7 and simulations using the best-fit model with N_{init}=300 and r_{sat}=402, corresponding to the experimental conditions (dilution starting at 5 x 10^5 cells ml^{-1} and reaching saturation at 2.01 x 10^8 cells ml^{-1} in average after 24h of growth). To match the experimental plots, simulated values are displayed at the same times as the experimental observations, i.e., once per day, from day 4 after tetrad dissection of* TLC1/tlc1Δ POL32/pol32Δ *diploids. Initial average telomere length was set to 300 bp, as expected in the initial diploid. Connecting lines correspond to the mean. Error bars of experimental values correspond to SD of the 4 independent experiments from reference 7. Error bars of simulated data correspond to SD of 30 independent simulations.* ***(d-e)*** *Population growth as a function of time* ***c*** *or as measured every 24h to match experimental conditions* ***d*** *for indicated r_{sat} values.*

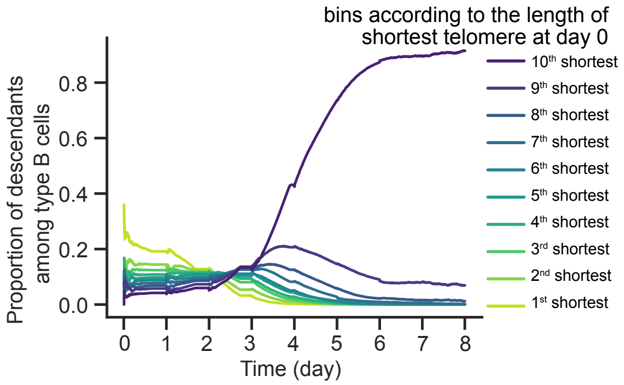

Supplementary Fig. 4: Additional hidden parameters of telomerase-deficient populations that are experimentally inaccessible. *Same as (Fig. 5c) for type B cells only.*

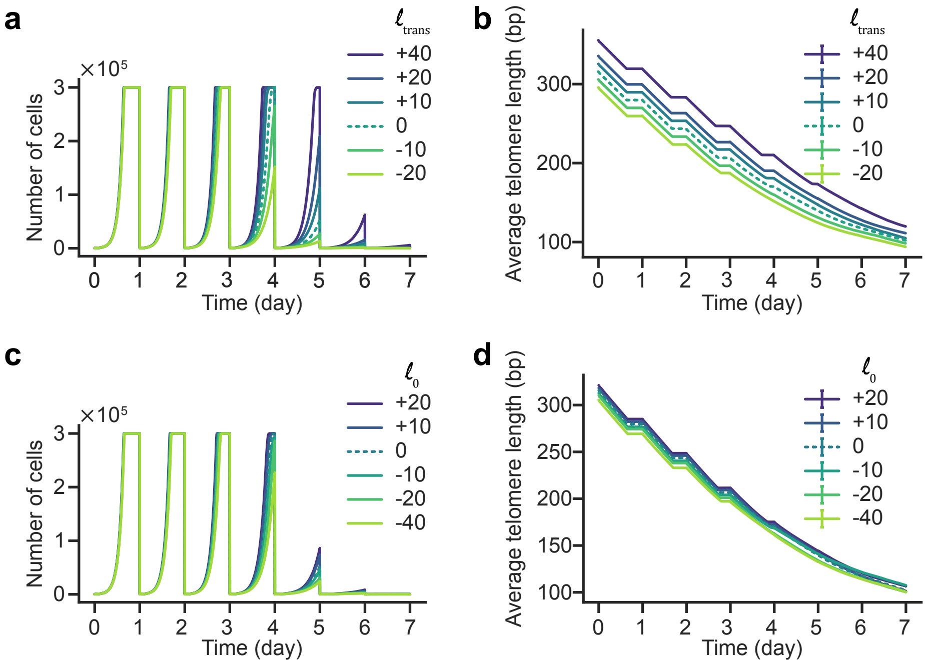

Supplementary Fig. 5: Effects on senescence rates from altering telomere length prior to telomerase inactivation a-d *Same as Fig. 6a-d but plots correspond to all values simulated with time (and not values reached each day).*

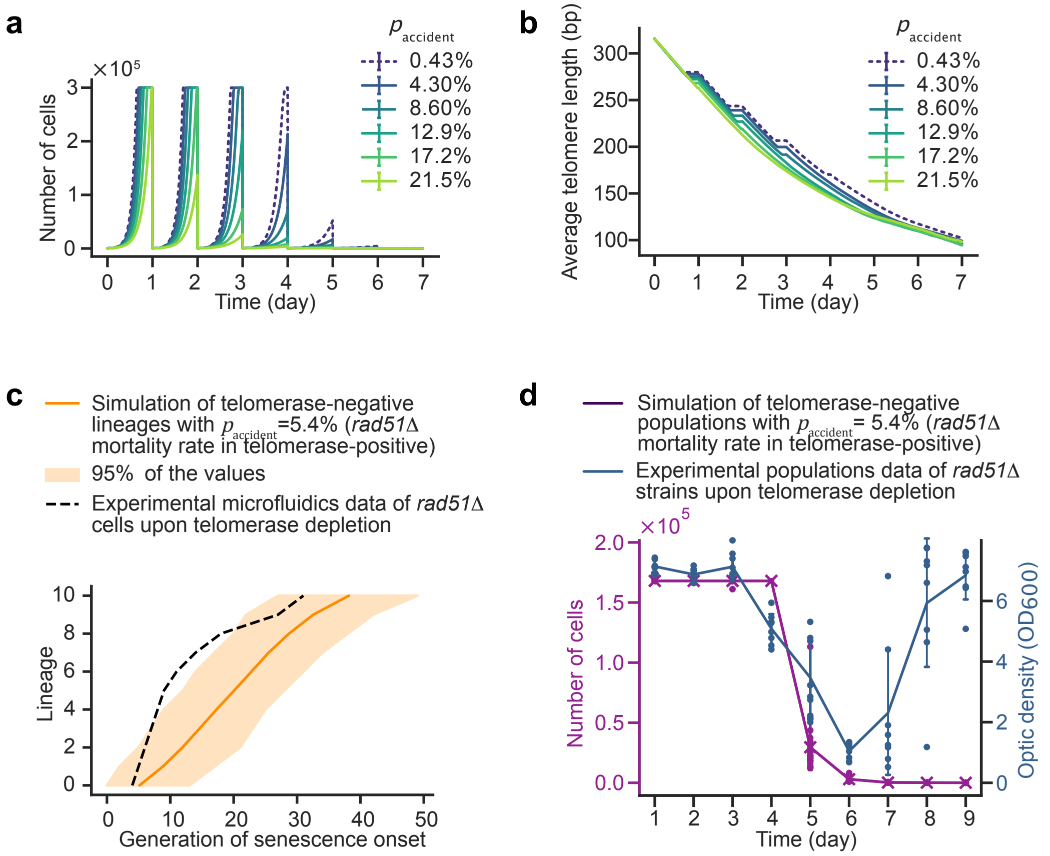

Supplementary Fig. 6: Effects on senescence rates from altering **telomere-independent spontaneous mortality rates** *($p_{accident}$)*. ***a-b*** *Same as Fig. 7a-b but plots correspond to all values simulated with time (and not values reached each day). (c) Lifespan of individual cell lineages of rad51Δ cells upon telomerase inactivation, as assessed by microfluidics (from [3]) and 1000 simulations of 11 individual cell lineages, corresponding the number of experimental lineages, using indicated $p_{accident}$. (d) Comparison between experimental data of populations of rad51Δ telomerase-negative cells and simulations using the best-fit model with $N_{init} = 300$ and $r_{sat} = 560$, corresponding to the experimental conditions (dilution starting at $OD_{600nm} = 0.0125$ and reaching saturation at $OD_{600nm} \approx 9$ after 24h in the absence of doxycycline (day 0). To match the experimental plots, simulated values are displayed at the same times as the experimental observations, i.e., once per day and $p_{accident}$ was taken as in (e). Connecting lines correspond to the mean. Error bars of experimental values correspond to SD of 8 independent experiments. Error bars of simulated data correspond to SD of 30 independent simulations.*

Supplementary References

1 Bourgeron, T., Xu, Z., Doumic, M. & Teixeira, M. T. The asymmetry of telomere replication contributes to replicative senescence heterogeneity. *Scientific reports* **5**, 15326 (2015). https://doi.org:10.1038/srep15326

2 Xu, Z., Duc, K. D., Holcman, D. & Teixeira, M. T. The length of the shortest telomere as the major determinant of the onset of replicative senescence. *Genetics* **194**, 847-857 (2013). https://doi.org:10.1534/genetics.113.152322

3 Xu, Z. *et al.* Two routes to senescence revealed by real-time analysis of telomerase-negative single lineages. *Nat Commun* **6**, 7680 (2015). https://doi.org:10.1038/ncomms8680

4 Coutelier, H. *et al.* The Polo kinase Cdc5 is regulated at multiple levels in the adaptation response to telomere dysfunction. *Genetics* **223** (2023). https://doi.org:10.1093/genetics/iyac171

Mathematical model linking telomeres to senescence in Saccharomyces cerevisiae reveals cell lineage versus population dynamics

Supplementary Methods on mathematical modeling, numerical simulation and parameter estimation

Anaïs Rat   Veronica Martinez Fernandez   Marie Doumic   Teresa Teixeira   Zhou Xu

December 20, 2024

In these Supplementary Methods, we provide all details on the mathematical model and the parameter inference.

1 Detailed description of the algorithm

Let us first sketch the main steps followed to simulate either the microfluidics or the population experiments; in the next sections we will give more details on each specific step. We use the same notations as in the Main Text and Methods.

1.1 Simulated microfluidics experiments

- Initial cell of state $\boldsymbol{S}^0 = (\boldsymbol{L}^0, T^0, C^0, \tau^0)$:
  - Telomere lengths $\boldsymbol{L}^0 \in \mathbb{N}^{2\times16}$ drawn as a 32− independent identically distributed sample according to f_{init} described in Section 2
  - Type $T^0 = A$,
  - Cell cycle type $C^0 = nor$,
  - Cell cycle duration τ^0 : random variable uniformly chosen from the experimental subdataset of normal cycles of *type A* shown in Supplementary Methods Fig. 1 (see also Fig. 1d for a kernel density smoothing representation)

- From one cycle S^k to the next S^{k+1}:
  - Draw a Bernoulli random variable of parameter $p_{accident}$ for accidental death; if yes, go to terminal step, if no, go to the next item.
  - Draw $\boldsymbol{L}^{k+1}$ according to the telomere shortening model described in Methods.
  - Compute $l^{k+1} = \min(L_1^{k+1}, \cdots, L_{32}^{k+1})$ the shortest telomere.
  - Draw C^{k+1} according to the following rule:
    * If $C^k = sen$, draw a Bernoulli random variable of parameter p_{death} for senescence death; if yes, go to terminal step, if no, take $C^{k+1} = sen$.
    * If $C^k \in \{nor, nta\}$, draw a Bernoulli random variable of parameter $p_{sen}(l^{k+1})$ for senescence: if yes, take $C^{k+1} = sen$, if no:
      · If $C^k = nor$, draw a Bernoulli random variable of parameter $p_{nta}(l^{k+1})$ for non-terminal arrest; if yes, take $C^{k+1} = nta$, if no, take $C^{k+1} = nor$.
      · If $C^k = nta$, draw a Bernoulli random variable of parameter p_{repair} to repair; if yes, take $C^{k+1} = nor$, if no, take $C^{k+1} = nta$.
  - Draw T^{k+1} according to the following rule:
    * If $T^k = B$ then $T^{k+1} = B$
    * If $T^k = A$ and $C^{k+1} \in \{nor, sen\}$ then $T^{k+1} = A$
    * If $T^k = A$ and $C^{k+1} = nta$ then $T^{k+1} = B$.
  - Cell cycle duration τ^{k+1} : random variable drawn uniformly from the experimental subdataset (see Supplementary Methods Fig. 1) which corresponds to the cell's type T^{k+1} and cell cycle type C^{k+1}.
- Terminal step: when for a given generation k^{last}
  - Accidental death: store $S^{k^{last}}$
  - Senescence death: if $T^{k^{last}} = A$, store $T^{k^{last}}$. If $T^{k^{last}} = B$ and the senescence occurs during a first series of non-terminal arrests, store $T^{k^{last}} = M$.

In practice, we store more variables than only S^k, in particular to keep the memory of non-terminal arrests.

1.2 Simulated population experiments

Populations are simulated along the same lines as for the microfluidic experiments, except that 1/ we keep the two daughter cells at division, 2/ we compute the time dynamics of the population, 3/ departing from a number of cells N_{init}, we stop the simulation as soon as either 24h is elapsed or when the cell count reaches $r_{sat} \times N_{init}$. We then pick at random N_{init} cells among the cells present at the time of saturation, and go through a new loop of simulation.

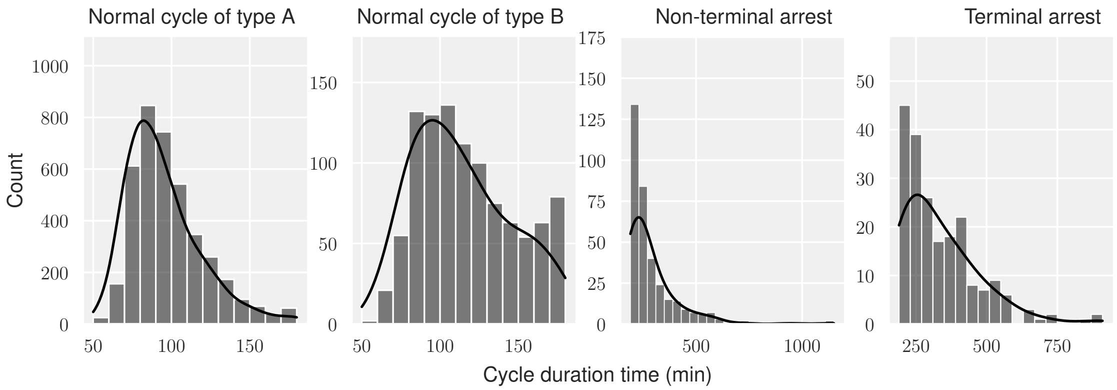

Supplementary Methods Fig. 1: Experimental distributions of cell cycle duration time among four distinct categories, defined on the basis of the experimental classification of lineages, for $D = 180\,\text{min}$. The set of terminal arrests plotted on these figures excludes the terminal arrests directly preceding death (which are significantly longer). The bin width is 10 min for normal cycle, 40 min for long cycles.

2 Initial distribution of telomere lengths

Departure point: a telomerase-positive telomere shortening/elongating model Given that generation 0 corresponds in our dataset to the inactivation of the telomerase, the initial distribution of telomere lengths corresponds to the distribution of telomere lengths in a telomerase-positive population of the same yeast strain as the dataset. It is natural to assume that in such a population, a steady distribution of telomere lengths has been reached, and is the same for any telomere of the cells. For this reason, we assume that initially all telomere lengths are independent, identically distributed (i.i.d.) according to the same law f_{init}:

$$L_{i,j}^{0} \sim f_{init}, \qquad i \in \{1, 2\}, \qquad j \in \{1, \ldots, 16\},$$

where $L_{i,j}^{0}$ is the random variable corresponding to the length of the ith telomere of the jth chromosome at generation 0.

To determine f_{init}, we thus rely on the distribution of telomere lengths f_0 of Bourgeron et al. [3] (Supplementary Fig. 1b) derived by adapting the numerical approach of [6] to the yeast strain that interests us. Their distribution approaches the stationary distribution of a certain Markov chain, whose parameters are fitted on experimental data, which describes telomere length shortening and elongation in the presence of telomerase.

Sensitivity analysis on the initial telomere length distribution Due to the fact that very few experimental measurements of the telomere length distribution f_0 are avail-

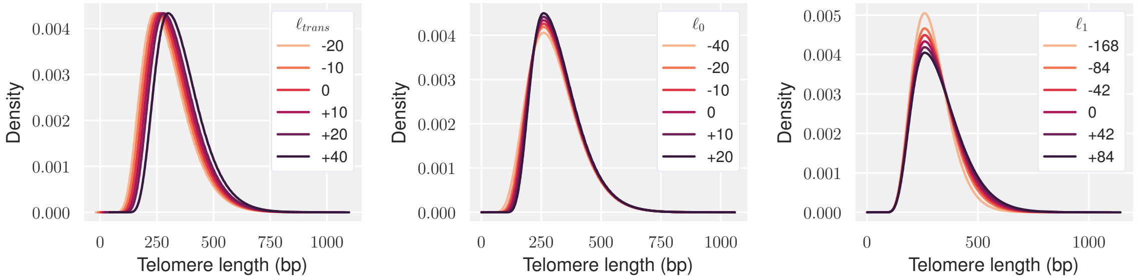

Supplementary Methods Fig. 2: Transformations of the distribution of telomere lengths f_0: translations *(left)*, dilation of the left tail *(middle)* and the right tail *(right)*.

able, so that it has been estimated indirectly from the mathematical shortening/elongation model described above and the experimental measurement of its statistical mode, we allowed f_{init} to deviate slightly from f_0, namely:

- Translations by ℓ_T.
- Dilations at both sides of the mode which preserve the (experimentally measured) mode:

$$\begin{cases} \inf\big(\mathrm{supp}(f_{init})\big) = \ell_{inf} + \ell_0 \\ \sup\big(\mathrm{supp}(f_{init})\big) = \ell_{sup} + \ell_1 \\ \mathrm{mod}(f_{init}) = \ell_{mode} \end{cases}$$

  to preserve the mode $\ell_{mode} := 260(16)\,\mathrm{bp}$ [7] of the experimental distribution and bring $[\ell_{inf}, \ell_{mode}]$ on $[\ell_{inf} + \ell_0, \ell_{mode}]$ and $[\ell_{mode}, \ell_{sup}]$ on $[\ell_{mode}, \ell_{sup} + \ell_1]$.

Finally, the transformation of f_0 can be written as

$$f_{init}\big(\cdot; \ell_T, \ell_0, \ell_1\big) = C \times \begin{cases} f_0\big((\ell - \ell_{inf} - \ell_0)\alpha_0 + \ell_{inf} - \ell_T\big) & \ell \in \big[\ell_{inf} + \ell_0, \ell_{mode}\big]\,, \\ f_0\big((\ell - \ell_{mode})\alpha_1 + \ell_{mode} - \ell_T\big) & \ell \in \big[\ell_{mode}, \ell_{sup} + \ell_1\big]\,, \end{cases} \tag{2.1}$$

with $C = C(\ell_0, \ell_1)$ a normalization constant, and

$$\alpha_0 := \frac{\ell_{mode} - \ell_{inf}}{\ell_{mode} - \ell_{inf} - \ell_0}, \qquad \alpha_1 := \frac{\ell_{sup} - \ell_{mode}}{\ell_{sup} + \ell_1 - \ell_{mode}}. \tag{2.2}$$

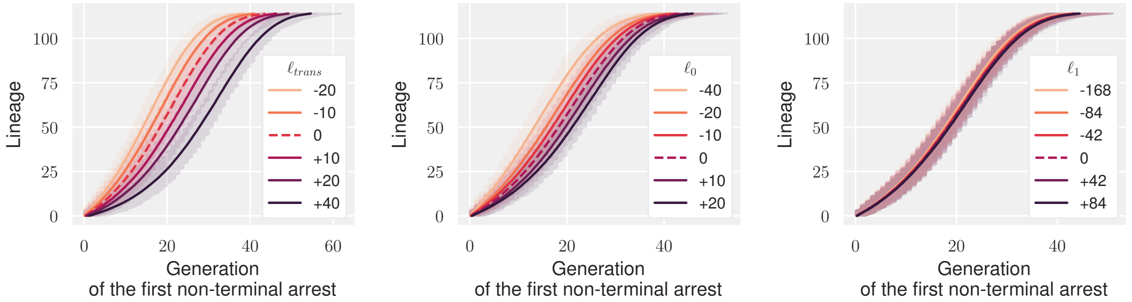

Supplementary Methods Fig. 3: Generation of the onset of the first non-terminal arrest with respect to the transformations of f_0 displayed in Supplementary Methods Fig. 2: translations *(left)*, dilation of the left tail *(middle)* and the right tail *(right)*. Average and 5th and 95th percentiles on $k = 1000$ simulations.

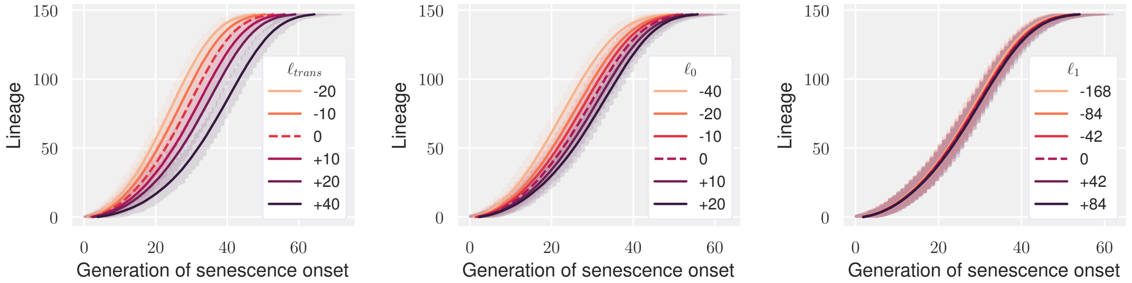

Supplementary Methods Fig. 4: Generation of the onset of senescence with respect to the transformations of f_0 displayed in Supplementary Methods Fig. 2: translations *(left)*, dilation of the left tail *(middle)* and the right tail *(right)*. Average and 5th and 95th percentiles on $k = 1000$ simulations.

In Supplementary Methods Fig. 2, modifications on f_{init} are displayed, and in Supplementary Methods Figs. 3, 4 their corresponding influence on the onset of non-terminal arrests (Supplementary Methods Fig. 3) and senescence (Supplementary Methods Fig. 4). The left tail of the distribution appears to have a great influence on the dynamics of microfluidics data - contrarily to its influence on the population experiment, see Main Text and Fig. 6a,c. We also see that the right tail dilation has only little influence.

The modifications of ℓ_0 and ℓ_1 are then fitted together with the laws of arrest on microfluidics data; the distribution f_{init} retrieved is plotted on Supplementary Fig. 1b.

3 Determination of N_{init}

In the population experiment, the initial concentration and the concentration of dilution are the same. Thus, if our simulations are initiated with N_{init} cells, dilution consists in

selecting N_{init} cells *uniformly* among the population reached before dilution.

As for saturation, we consider that when the number of individuals becomes too high, all individuals stop dividing/evolving. To reach saturation we fix a population size N_{sat} at which the population saturates. Since we cannot reasonably reach the experimental concentration *in silico* we rather fix the ratio r_{sat} such that $N_{sat} = r_{sat} N_{init}$. In the experimental dataset $r_{sat} \approx 720$ whereas in other experiments it may be much larger.

Representativeness analysis – sensitivity to $\mathbf{N_{init}}$. We compare the behavior of populations originating from *different initial number of cells* N_{init}. In order to accurately estimate these behaviors (i.e. to have an empirical behavior close to the statistical one) we simulated $k = 25$ times the evolution of a population with a certain fixed initial size N_{init}.

Variability decreases with N_{init} up to a certain point. The first, expected, observation is that *the less cell initially present, the more variability between simulations*, see Supplementary Methods Figs. 5, 6, 8, 10, 11 and Supplementary Table 1. This supports the idea that the variability in the distribution of initial telomere lengths is an important source of heterogeneity in senescence [1, 6]. The decrease in variability however seems to stabilize around $N_{init} = 200$ around a value that should correspond to the variability intrinsic to the stochastic evolution.

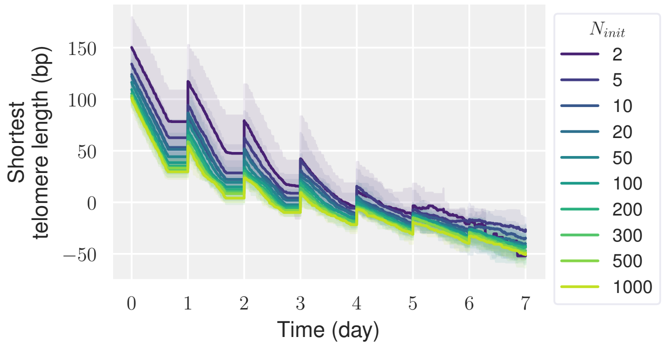

Supplementary Methods Fig. 5: Time evolution of the minimal telomere length in the population with respect to the initial number of cells N_{init}. Average and 5th and 95th percentiles on $k = 25$ simulations. (The plateau sections correspond to saturation, during which the population stops evolving.) Although decreasing with N_{init}, the shortest-telomere length seems to converge.

Extreme behaviors are sensitive to N_{init} unlike average behaviors. Starting initially with more cells, we are more likely to hit the tails of the distribution of initial telomere lengths and to have chromosome shortening mainly on the same extremity, resulting in a

fast shortening telomere and a slow shortening one. Extremum quantities should thus be sensitive to N_{init}.

This can be observed for example looking at the evolution of the minimum telomere length in the population plotted on Supplementary Methods Fig. 5.

The same reasoning applies to the extinction time of the population, which is nothing other than the maximal lineage lifetime in the population: it keeps increasing with the initial number of cells. And similarly with the senescing time, see Supplementary Methods Fig. 6.

Since the length of the shortest telomere is a major determinant of the onset of senescence, Supplementary Methods Figs. 7, 6 can be partially explained looking at the distributions f^n_{min} and $f^n_{min-max}$ of the minimum and maximum, respectively, of the shortest telomere in a population of n cells (each cell possessing 32 telomeres). As shown on Supplementary Methods Fig. 7, they get translated and narrowed for large n. To access f^n_{min} and $f^n_{min-max}$ we compute their cumulative distribution functions, taking advantage of the independence of telomere lengths at generation 0:

$$\begin{aligned} F^n_{min}(\ell) &= \mathbb{P}\Big(\min_{1\leq i\leq n}\Big(\min_{1\leq j\leq 32} L_{i,j}\Big) \leq \ell\Big) = 1 - \mathbb{P}\Big(\bigcap_{\substack{1\leq i\leq n\\ 1\leq j\leq 32}} L_{i,j} > \ell\Big) \\ &= 1 - \big(1 - F_{init}(\ell)\big)^{32n} \end{aligned}$$

where F_{init} denotes the cumulative distribution function of f_{init}, and

$$\begin{aligned} F^n_{min-max}(\ell) &= \mathbb{P}\Big(\max_{1\leq i\leq n}\Big(\min_{1\leq j\leq 32} L_{i,j}\Big) \leq \ell\Big) = \mathbb{P}\Big(\bigcap_{1\leq i\leq n}\Big(\min_{1\leq j\leq 32} L_{i,j}\Big) \leq \ell\Big) \\ &= F^1_{min}(\ell)^n = \Big(1 - \big(1 - F_{init}(\ell)\big)^{32}\Big)^n, \end{aligned}$$

from which we deduce

$$\begin{cases} f^n_{min}(\ell) = F^n_{min}(\ell) - F^n_{min}(\ell - 1) = \big(1 - F_{init}(\ell - 1)\big)^{32n} - \big(1 - F_{init}(\ell)\big)^{32n}, \\ f^n_{min-max}(\ell) = \Big(1 - \big(1 - F_{init}(\ell)\big)^{32}\Big)^n - \Big(1 - \big(1 - F_{init}(\ell - 1)\big)^{32}\Big)^n. \end{cases} \tag{3.1}$$

Expectedly, the evolution of average indicators are less sensitive to N_{init} than the time evolution of extrema, as for example:

- The average generation (Supplementary Methods Fig. 8-*right*).
- The average over all cells of the average telomere length, or even of the shortest telomere length. (Supplementary Methods Fig. 8-*left*).

These are quantities that vary less with respect to n.

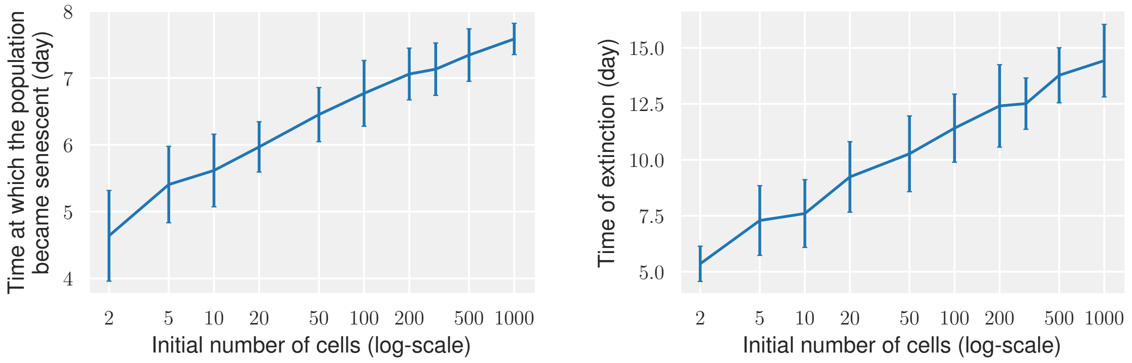

Supplementary Methods Fig. 6: Graphs of the times of senescence *(left)* and extinction *(right)* of simulated populations with respect to the initial number of cells N_{init}. Average and standard deviation on $k = 25$ simulations. Although senescence and extinction times keep increasing with N_{init}, the log-scale indicates that the increase tends to zero.

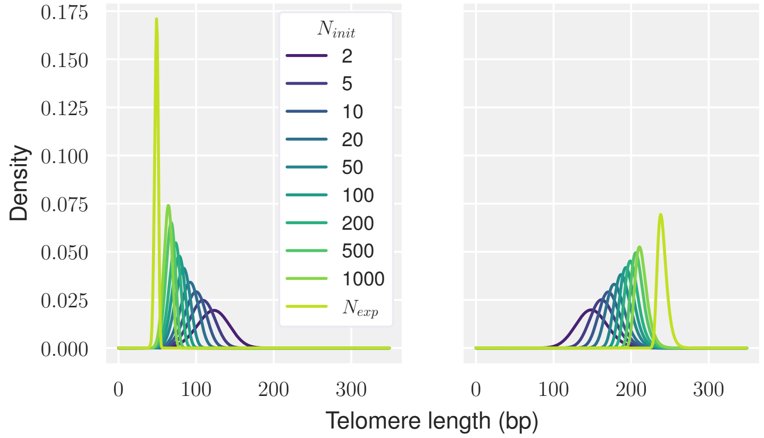

Supplementary Methods Fig. 7: Distribution of the minimal *(left)* and maximal *(right)* shortest telomere length in a population of N_{init} cells.

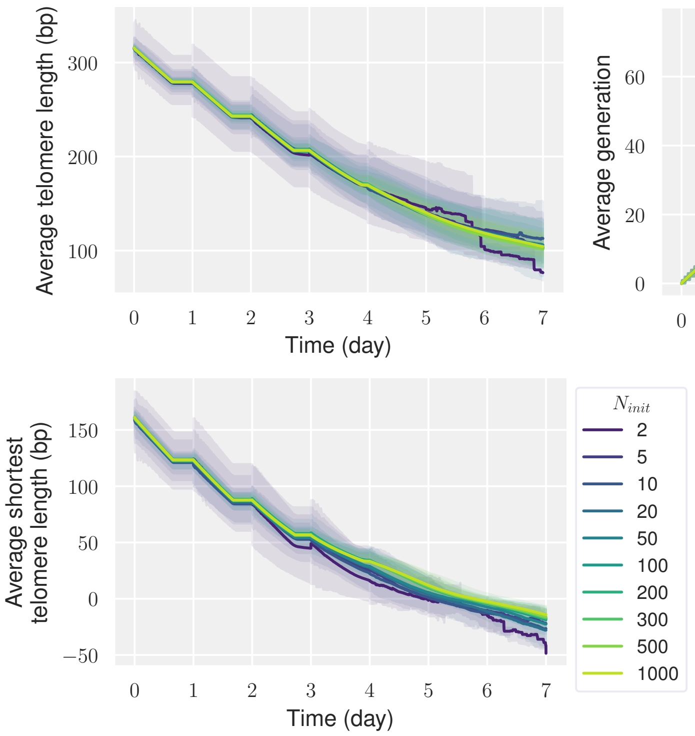

Supplementary Methods Fig. 8: Time evolution of three population average quantities with respect to the initial number of cells N_{init}: the generation *(right)*, cells' average *(top left)* and shortest *(bottom left)* telomere length. Average (and 5th and 95th percentiles at the left) on $k = 25$ simulations.

Saturation bias. Likewise, another time indicator, more relevant than the extinction time since less sensitive to the extreme behaviors, is the time when the saturation limit is reached (in the Main Text we also compare, during the sensitivity analysis, the time when half of the saturation limit is reached (HSL)). This saturation time is directly linked to the doubling-time of the population. On Supplementary Methods Fig. 9 we see that it stabilizes in average for N_{init} greater than 50 and in standard deviation around $N_{init} = 200$, in accordance with Supplementary Table 1.

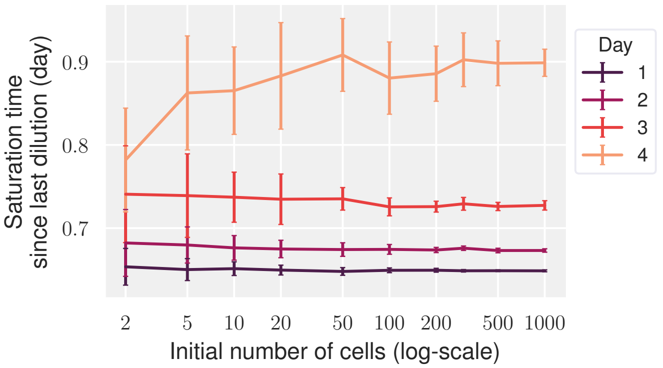

Supplementary Methods Fig. 9: Graph of the times of saturation (on each day of saturation) of simulated populations with respect to the initial number of cells N_{init}. Average and standard deviation among the simulations that have reached saturation among $k = 25$ simulations.

$\mathbf{N_{init}}$	**2**	**5**	**10**	**20**	**50**	**100**	**300**	**500**	**1000**
**Day 1**	1	1	1	1	1	1	1	1	1
**Day 2**	1	1	1	1	1	1	1	1	1
**Day 3**	0.84	0.96	1	1	1	1	1	1	1
**Day 4**	0.24	0.44	0.4	0.48	0.72	0.96	1	1	1

Supplementary Methods Table 1: Table of the proportion of simulations (among $k = 25$ simulations) that have saturated per day with respect to the initial number of cells N_{init}.

Having a closer look, we notice that not only small populations die and senesce earlier than large ones (Supplementary Methods Fig. 6), but they also saturate slightly later on the first days of saturation and they saturate "less" on the third and fourth day than large populations (Supplementary Methods Figs. 9, 10, Supplementary Table 1). In addition, the few small populations that have saturated on the fourth day have saturated earlier than large populations.

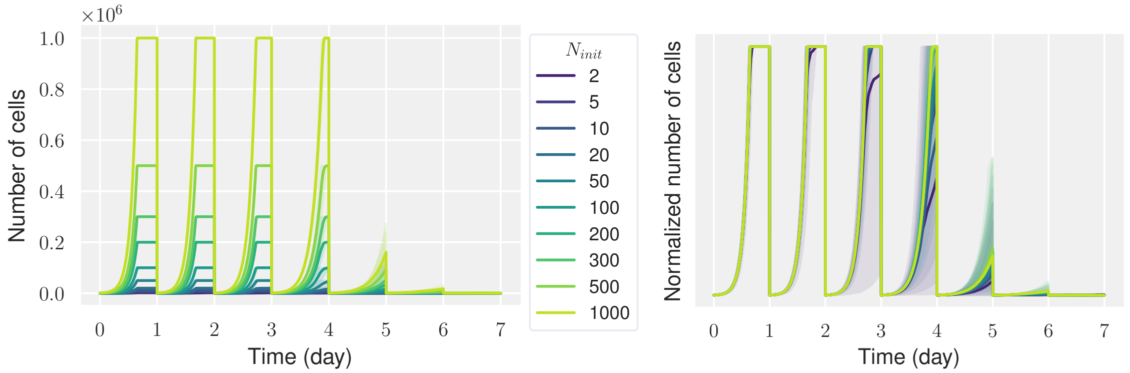

Supplementary Methods Fig. 10: Time evolution of the number of cells with respect to the initial number of cells N_{init}. The number of cells through time is the effective one at the *(left)* and it is renormalized (by $\frac{N_{init}}{500}$) at the *(right)*. Average and 5th and 95th percentiles on $k = 25$ simulations.

Let us explain these observations. Given our model, the evolution of cells is independent from each other. Under growth, division and death processes, any given subpopulation is therefore evolving independently from the rest of the population. However, cells become coupled through the total number of cells as soon as we add saturation at threshold N_{sat} (all the cells stop evolving as soon as the total number of cells reaches N_{sat}). A population with a small pool of fast/slow dividing lineages will then reach saturation –and thus stop evolving– earlier/later than an homogeneous population, with the consequence of decreasing/increasing in the other part of the population the average generation at the next dilution.

This explains why most of the small populations, by saturating late on the two first days, avoid saturation on the following days. Because cells have stopped evolving later, they are "closer to death" in accordance with early extinction time, and high proportion of senescent cells compared with large populations (see Supplementary Methods Fig. 11-*right*). On the contrary, the few small populations that have saturated on the 3rd and 4th days, the same that managed to reach day 7, are made of lineages that have undergone arrest: the proportion of *type B* cell on the last day (an average only among non-extincted populations) is particularly high (Supplementary Methods Fig. 11-*left*) and the average generation particularly low (Supplementary Methods Fig. 8-*right*).

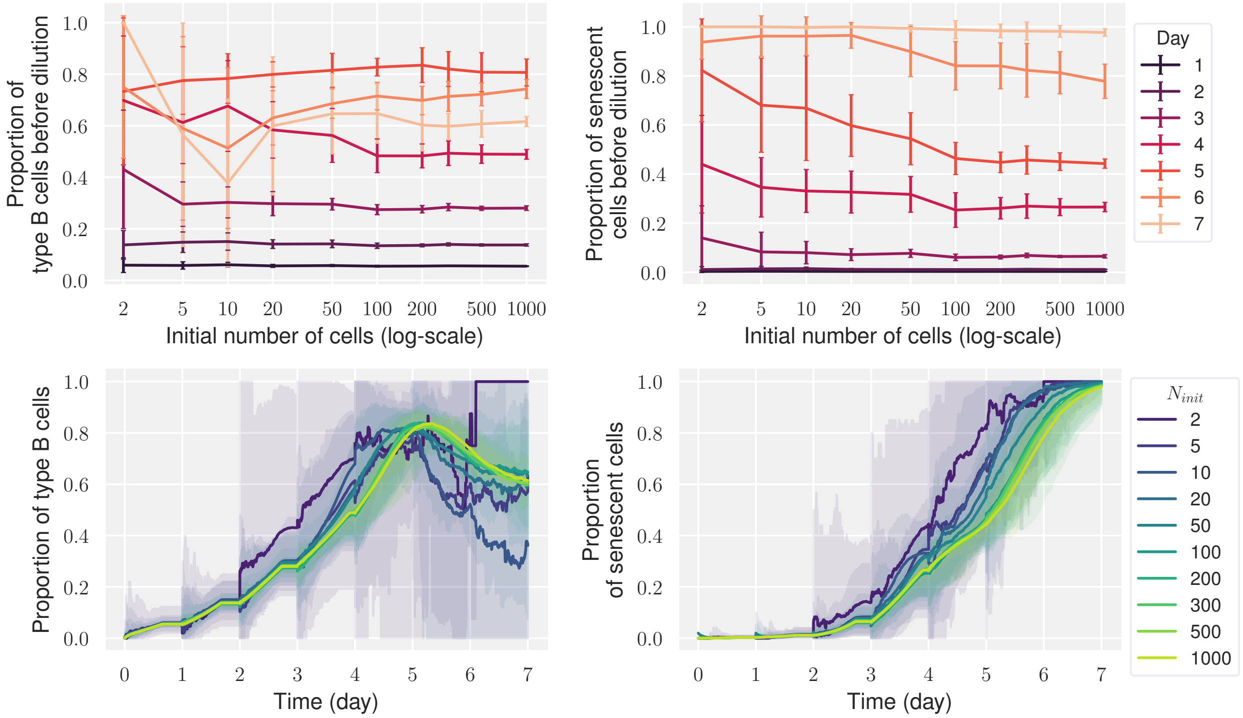

Supplementary Methods Fig. 11: Proportion of *type B* cells *(left)* and senescent cells *(right)* in the population before each dilution *(top)* or at each time of the experiment *(bottom)* with respect to the initial number of cells N_{init}. Average and standard deviation *(top)* or 5th and 95th percentile *(bottom)* on $k = 25$ simulations. One should be careful that at each time, these statistics are actually taken only on those of the k simulations that are not extincted at this time, which bias the graphs of small populations at large times, made as an average on very few simulations.

Dilution bias. Such a saturation bias is accentuated by dilution since a small pool of fast/slow dividing lineages will be over/under represented right before dilution. Therefore

the rest of the population will have less/more chance to be selected by dilution than it had been in an homogeneous population.

The dilution bias should be significant only for the (small) populations that present a lot of variability in the saturation time. As already noticed, starting from around $N_{init} = 200$ the initial population is large enough to have small fluctuations of the saturation time.

4 Implementation and description of the code

The implementation of the model was coded in the Python programming language (Python Software Foundation, https://www.python.org/), with 3.8 Python version. The whole project is available at https://github.com/anais-rat/telomeres.

Architecture of the code

The python project is composed of 4 directories:

- `data`. Contains all the raw experimental data needed to run the simulations and compare them with experiment. It should not be modified unless you want to test the code and run simulations on new data. The GitHub folder contains already processed data in the subdirectory `processed`, which is obtained by running the script `makeFile/process_dataset.py`.
- `main`. Contains two subdirectories, `lineage` and `population`, for running lineage and population simulations, respectively. This is the directory where the main codes to run are gathered.
- `makeFile`. Contains the scripts that should be run to posttreat the raw data, convert it into a format recognized by the code and saved it in the `data/processed` directory. These scripts should be run before the simulations if the `data/processed` directory does not exist or is not up to date.
- `telomeres`. This package contains all the necessary auxiliary codes. The scripts in this folder are not intended to be modified or used directly to run simulations.

When running the "main" codes (i.e. those contained in `main`), they create new directories containing simulation results in `.npy` files and/or figures. For instance, the code `main.lineage.plot.py` will create folders and figures in the directory `figures`.

5 Inference of the parameters with microfluidics data

In the present section we present how we fit the parameters of the laws p_{nta}, p_{sen_A} and p_{sen_B} that characterize the onset of non-terminal and terminal arrests, respectively, en-

abling small transformations of the distribution f_0 of initial telomere lengths. We rely on microfluidic data and the simulation of lineages.

5.1 Description of the estimation method

Experimental data to fit. The experimental data upon which to base the estimation should highly depend, in our model, on the parameters to estimate. We thus consider the data plotted on Supplementary Methods Fig. 12-*left* that represent:

1. G_{sen} the generations at which senescence occurred, in the $n_{sen} = 148$ *experimentally senescent*, *i.e.* terminated by a long cycle and death, lineages of the dataset.

2. G_{sen_A} the subset of the generations G_{sen} of senescence made only of the $n_{sen_A} = 64$ (dead senescent) lineages classified as *experimental type A*. These are the lineages composed uniquely of normal cycles and experimental senescence.

3. G_{sen_B} similarly with the remaining $n_{sen_B} = 84$ lineages of *experimental type B*. These are lineages presenting at least one arrest followed by a normal cycle.

4. G_{nta} the generations at which occurred a first non-terminal arrest, generated from a total of $n_{nta} = 115$ experimental *type B* lineages.

5. The proportion of the experimentally senescent lineages classified as *type B*:

$$r_{B\%} = 100 \times \frac{n_{sen_B}}{n_{sen}} \approx 57\,\%.$$

Notations. In the following $G_{sen}(j)$ denotes the generation of senescence onset in the jth senescent lineage of the dataset (when ordering by increasing generation of senescence) and similarly with the types of arrest ("nta", "sen_A" and "sen_B"). Therefore the maps plotted on Supplementary Methods Fig. 12-left correspond to G^{-1}, but we abusively call them G-graphs (or generation graphs*). Wee keep this choice by sake of coherence with the previous articles* [3, 4].

Simulated "generation graphs". To these G-graphs actually corresponds the theoretical graphs given by our model, say $\hat{G}$. We approach them with Monte Carlo estimators $\hat{G}^{(k)}$, realized as an average on k independent simulated graphs $(\hat{G}^1, \ldots, \hat{G}^k)$:

$$\hat{G}_i^{(k)} : j \mapsto \frac{1}{k} \sum_{s=1}^{k} \hat{G}_i^s(j), \qquad i \in \{sen,\ sen_A,\ sen_B,\ nta\},\ j \in \{1, \ldots, n_i\}. \tag{5.1}$$

We make sure that each of the k simulated graphs presents the same characteristics as the associated experimental graph: it is made of the same number of generations, similarly ordered and with same characteristics (i.e. describing the onset of a specific type of arrest in a specific type of lineage, as perceived experimentally).

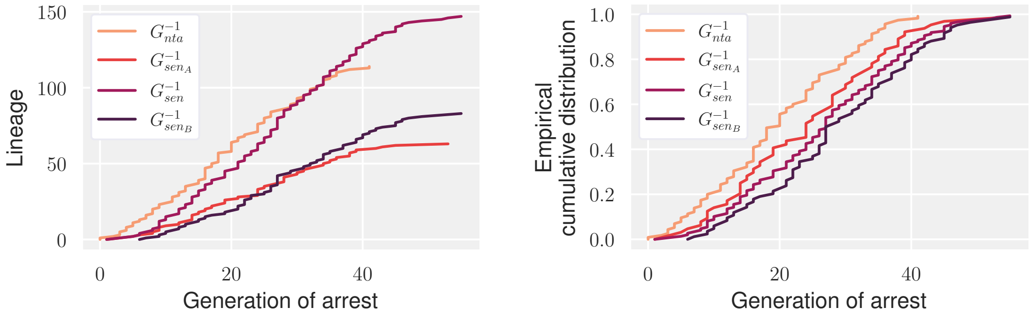

Supplementary Methods Fig. 12: Representation of the experimental generations (x-axis) at the onset of a first non-terminal (nta) or terminal (sen) arrest, among different lineages (indexed on the y-axis by increasing generation of arrest) of same characteristics (classified as A or B *type* and/or senescent) *(left)*. They are obtained from the lineage dataset (see Fig. 1c of the Main Text) after classifying every cell cycle as either long or normal with threshold $D = 180\,\text{min}$. Every lineage is classified depending on the location of its long cycles and we extract the generation(s) at which it got arrested. For every generation graph, renormalizing the y-axis yields the empirical cumulative distribution function of the experimental law of the associated generation of first arrest *(right)*.

Dependencies between simulated graph and parameters. A delicate point is that most of this data depends on both the law for non-terminal arrests and senescence, and slightly on p_{repair}.

In our model, we test at each division whether or not a newborn cell enters senescence (if its mother is not senescent) or enter or exit a non-terminal arrest. In particular, to enter a first non-terminal arrest at generation g, a lineage must have reached generation g without having undergone any arrest. Thus, $\hat{G}_{nta}$ depends not only on the probability to experience a first non-terminal arrest but also on the probability to enter senescence.

Likewise, for a fixed law of senescence onset, if for example p_{repair} is small and p_{nta} is such that the entry in senescence is much more likely than the entry in a sequence of non-terminal arrest(s), very few lineages will have time to enter and exit a non-terminal sequence of arrests before entering senescence, with great impact on the proportion of experimental *type* B lineages and the generation graphs.

This last bias is mainly due to the fact that, in order to generate the graphs of generations approaching the experimental graphs, the generations of arrest and thus the type of lineages are as they would have been perceived experimentally rather than as they are in the model. For our best-fitted parameters, around 16 % of senescent lineages are misclassified *type* A which is not negligible.

Parameters to estimate. Even though the parameters of p_{nta} were fitted by Martin et al. [4] with the same dataset, they were fitted with a fixed law of senescence entry,

consisting in a deterministic threshold at 0 bp. Because of the dependency of the simulated graphs on both laws, we need to estimate p_{nta} again, simultaneously with p_{sen_A} and p_{sen_B}.

Still, we do not re-estimate p_{repair}, the probability to exit a non-terminal arrest given that senescence is not triggered. It was fitted by Martin et al. [4] with the distribution of the number of consecutive non-terminal arrests among all the sequences of non-terminal arrests. We believe indeed that modifying their laws of arrest in a way that fits the experimental curves should not modify much the distribution of consecutive long cycles (and thus p_{repair}). We verified this a posteriori with the best-fit laws of arrest.

Method. We used the evolutionary algorithm CMA-ES in its 3.2.2. python version available on GitHub at https://github.com/CMA-ES/pycma. Based on the principles of biological evolution, CMA-ES explores in a stochastic and evolutionary-driven way a certain domain $\mathcal{D}$ of parameters in order to estimate the point(s) of the domain that minimizes a given cost function E [2]. For more details on the algorithm and on the CMA-ES parameters that we have adjusted see Supplementary Table 2 or directly the GitHub link.

The cost function is defined as some weighted sum of the errors, in ℓ^2-norm, between the four experimental G-graphs[1] and their estimators $\hat{G}^{(k)}$ (defined by (5.1)) obtained from k simulations with the parameters $p \in \mathcal{D}$:

$$E_\omega(p) := \sum_{i \in \mathrm{I}} \omega_i \left\| G_i - \hat{G}_i^{(k)}(p) \right\|_{\ell^2}, \qquad \mathrm{I} = \{sen,\, sen_A,\, sen_B,\, nta\}, \tag{5.2}$$

Similarly, we denote by

$$e_{B\%}(p) := \left| r_{B\%} - \hat{r}_{B\%}^{(k)}(p) \right|, \qquad \hat{r}_{B\%}^{(k)}(p) := \frac{1}{k} \sum_{s=1}^{k} \hat{r}_{B\%}^{s}(p), \tag{5.3}$$

the error (in absolute value) between experimental and estimated proportions of *type B* lineages among n_{sen} senescent lineages; with $\hat{r}_{B\%}^{(k)}(p)$ the estimator of the proportion defined as the average on k independent simulations with parameters p.

The parameters of these error functions are listed in Supplementary Table 2 and the different parameter domains $\mathcal{D}$ that have been explored are explicitly given in Supplementary Table 3 of Section 5.4. We denote by $\mathcal{D}_{m,i}$ the ith domain of dimension m and by $\mathcal{D}_m$ the union of all the $\mathcal{D}_{m,i}$, $i \geq 1$, introduced.

5.2 Characterization of the estimation method

CMA-ES estimation strategies and robustness. Overwhelming empirical evidence agree on CMA-ES convergence for most cost functions, and first theoretical grounds may

[1] See notations in the first paragraph of Section 5.1. One should not be mistaken: on the "G-graphs" plotted on Supplementary Methods Fig. 12-*left* the distance is not taken "horizontally" as usual, but "vertically" since the distance is actually between G^{-1} maps.

		**Parameter**
**CMA-ES**	N	*Population size.* The number of points –in the parameter space $\mathcal{D}$– tested (i.e at which the cost function is evaluated) per iteration, or "generation". At every iteration i, the N new points of the ith generation are drawn from a normal distribution with parameters (p_i, Σ_i) depending on the value of the cost function at the N points of the previous generation. Here $p_i \in \mathcal{D}$ is the approximation of the solution at generation i, expected to converge to a minimizer of E while Σ_i would get closer to 0_N.
	p_0	*Starting point.* The N points of the generation 0 are drawn from a normal distribution of parameters $(p_0, \sigma_0 D)$, with D is the identity N-matrix if $\mathcal{D} = [0,1]^m$, and some rescaled (diagonal) N-matrix otherwise.
	σ_0	*Initial standard deviation.* See above. It should be small if p_0 is *a priori* close to the solution.
**Error functions**	ω	*Weights* of the errors from the different graphs, see (5.2)
	$\bar{e}_{B\%}$	*Upper bound for $e_{B\%}$.* For every candidate $p \in \mathcal{D}$, if it is such that the error $e_{B\%}(p)$ (defined by (5.3)) exceeds $\bar{e}_{B\%}$, the value of the cost function at p is set to `NaN` which forces CMA-ES to forget p and draw another point. At the end the optimization is run in the subset of $\mathcal{D}$ where $e_{B\%} \leq \bar{e}_{B\%}$. This is a good way to avoid runtime errors in the simulation of generations arrest of *type A* or B lineages in subsets of $\mathcal{D}$ corresponding to laws of arrest that makes *type A* or B lineages very unlikely (where the proportion of *type B* lineages close to 1 or 0, resp.).
	k	*Number of simulations of experimental G-graph* to run and average to approximate the corresponding theoretical graph.
	$\lVert\cdot\rVert_{\ell^2}$	*Distance* defining the error between experimental and simulated graphs: $$\lVert G \rVert_{\ell^2} := \sqrt{\sum_{l=1}^{n} \lvert G(l) \rvert^2}, \qquad G \in \mathbb{R}^n$$ NB: we have $n_{nta} = 115$, $n_{sen} = 148$, $n_{sen_A} = 64$ and $n_{sen_B} = 84$.

Supplementary Methods Table 2: Table of the parameters of the optimization method, distinguishing between parameters of the optimizer (CMA-ES) and of the error functions (E and $e_{B\%}$).

be found in [5]. To optimize the estimation strategy and reinforce CMA-ES robustness, we followed two classical recommendations:

- Several estimations were run in series: each new simulation kept track of the solutions returned by the previous estimations and started with bigger initial *population sizes* (defined in Supplementary Table 2). In this case, we denote $N = (N_1, N_2, \dots, N_l)$ the l successive *population sizes.*
- Every estimation strategy was launched several times in parallel with exact same setting but different *starting point* p_0 (again we refer to Supplementary Table 2) drawn independently in the parameter space. At the end, we checked that most of the solutions obtained were close to each other.

Sensitivity to the weight ω. To reinforce the robustness of our results we performed a sensitivity analysis to the weight ω defining the cost function (5.2), running independent CMA-ES optimizations for various ω. The results, gathered in Section 5.4, suggest that the optimization is not very dependent on ω. In the following we thus chose $\omega \coloneqq (0.3, 1, 1, 1)$ which brought the best results.

5.3 A few comments on the proportion of *type B* cells

Contrarily to the generations of arrest, the proportion of *type B* lineages obtained with our laws ($\hat{r}_{B\%} \approx 74\,\%$) is slightly too high compared with the experimental value ($r_{B\%} \approx 57\,\%$). Although the discrepancy is significant, it must be qualified by the following considerations:

- *The experimental value is likely biased downwards.* Because of how is defined the classification of lineages, experimental type A are necessarily senescent dead lineages ($n_A = n_{sen_A}$), contrarily to type B ($n_B = n_{nta} \neq n_{sen_B}$). However not all the $n = 187$ lineages of the dataset have reached senescence: $39 = n - n_{sen}$ have not, because dead accidentally or still alive at the end of the experiment. Among them 31 ($= n_B - n_{sen_B}$) identify as type B, while only $n_X = 8$ are unclassified. As a result, there are proportionally more type B lineages than type A in the whole dataset than in the subdataset of senescent lineages:

$$\bar{r}_{B\%} \approx 100 \times \frac{n_B}{n_A + n_B} \approx 64\,\%, \qquad \bar{r}_{B\%} \in \left[\bar{r}_{low}, \bar{r}_{up}\right],$$
$$\bar{r}_{low} \coloneqq 100 \times \frac{n_B}{n_B + n_A + n_X} \approx 61\,\%, \qquad \bar{r}_{up} \coloneqq 100 \times \frac{n_B + n_X}{n_B + n_A + n_X} \approx 67\,\%,$$

  with $\bar{r}_{B\%}$ be the proportion of type B lineages in the whole dataset.

- *The confidence interval is sufficiently large.* The 5th and 95th percentiles of a k-sample, for $k = 1000$ simulations, of the proportion $\hat{r}_{B\%}$ given by our laws are around $67\,\%$ and $80\,\%$, and the extrema around $61\,\%$ and $89\,\%$.

These two points reconcile the discrepancy between the experimental and simulated proportions of type B lineages, since $\bar{r}_{B\%}$ belong to the (empirical) 90 % confidence interval given by our laws.

5.4 More details on the estimations carried out

Let us recall the probability laws whose parameters were estimated.

- The probability for the shortest telomere of length ℓ to trigger a first sequence of non-terminal arrest(s):

$$p_{nta}(\ell) = \min\bigl(1, b_{nta}\, \mathrm{e}^{-a_{nta}\ell}\bigr), \quad (a_{nta}, b_{nta}) \in (0,1]^2. \qquad (\mathcal{P}_{nta})$$

- The probability for a cell with shortest telomere of length ℓ to enter senescence when it is assumed independent of cell type: for $(a_{sen}, b_{sen}, \ell_{min}) \in [0,1] \times (0,1] \times \mathbb{Z}$,

$$p_{sen}(\ell) = \begin{cases} \min\bigl(1, b_{sen}\, \mathrm{e}^{-a_{sen}\ell}\bigr), & \text{if } \ell > \ell_{min}, \\ 1, & \text{if } \ell \leq \ell_{min}. \end{cases} \qquad (\mathcal{P}_{sen})$$

- The previous law when we assume that it is different for *type A* and *type B* cells:

$$p_{sen_A}(\ell) = \begin{cases} \min\bigl(1, b_{sen_A}\, \mathrm{e}^{-a_{sen_A}\ell}\bigr), & \text{if } \ell > \ell_{min_A}, \\ 1, & \text{if } \ell \leq \ell_{min_A}, \end{cases} \qquad (\mathcal{P}_{sen_A})$$

$$p_{sen_B}(\ell) = \begin{cases} \min\bigl(1, b_{sen_B}\, \mathrm{e}^{-a_{sen_B}\ell}\bigr), & \text{if } \ell > \ell_{min_B}, \\ 1, & \text{if } \ell \leq \ell_{min_B}. \end{cases} \qquad (\mathcal{P}_{sen_B})$$

In addition, we fitted the parameters ℓ_{trans}, ℓ_0 and ℓ_1 of the initial distribution of telomere lengths. We refer to (2.1) for their definition.

The different domains on which estimations have been run are described on Supplementary Table 3. We denote by $\mathcal{D}_{m,i}$ the ith domain of dimension m and by $\mathcal{D}_m$ the union of all the $\mathcal{D}_{m,i}$, $i \geq 1$, introduced.

Sensitivity to the weight ω

To reinforce the robustness of our results we run CMA-ES optimization strategies on the two domains $\mathcal{D}_{5,1}$ and $\mathcal{D}_{8,2}$ for different weights ω defining the cost function (5.2). We tested weights of the form ω^i and $\omega^i \times \omega^\ell$, $i \in \{1,2,3,4\}$ before renormalization, see Supplementary Table 4. The results are gathered in Supplementary Table 5 and Supplementary Methods Fig. 13.

Domain	Parameter range					
	$(\mathcal{P}_{nta})$		$(\mathcal{P}_{sen})$			f_0
	a_{nta}	b_{nta}	a_{sen}	b_{sen}	ℓ_{min}	ℓ_0
$\mathcal{D}_{5,1}$	$[0,1]$	$[0,1]$	$[0,1]$	$[0,2]$	$[\![0,80]\!]$	$\{0\}$
$\mathcal{D}_{5,2}$	$[0,1]$	$[0,1]$	$[0,1]$	$[0,1]$	$[\![20,80]\!]$	$\{0\}$
$\mathcal{D}_{6}$	$[0,1]$	$[0,1]$	$[0,1]$	$[0,1]$	$[\![27,40]\!]$	$[\![0,30]\!]$

(a) Senescence entry assumed identical for *type A* and *type B* cells

Domain	Parameter range										
	$(\mathcal{P}_{nta})$		$(\mathcal{P}_{sen_A})$			$(\mathcal{P}_{sen_B})$			f_0		
	a_{nta}	b_{nta}	a_{sen_A}	b_{sen_A}	ℓ_{min_A}	a_{sen_B}	b_{sen_B}	ℓ_{min_B}	ℓ_{trans}	ℓ_0	ℓ_1
$\mathcal{D}_{7}$	$[0,1]$	$[0,1]$	$[0,1]$	$[0,1]$	$\{30\}$	$[0,1]$		$[\![0,40]\!]$	$\{0\}$	$\{0\}$	$\{0\}$
$\mathcal{D}_{8,1}$	$[0,1]$	$[0,1]$	$[0,1]$	$[0,2]$	$[\![0,80]\!]$	$[0,1]$	$[0,2]$	$[\![0,80]\!]$			
$\mathcal{D}_{8,2}$	$[0,1]$	$[0,1]$	$[0,1]$	$[0,1]$	$[\![20,80]\!]$	$[0,1]$	$[0,1]$	$[\![0,60]\!]$			
$\mathcal{D}_{8,3}$	$[0,1]$	$[0,1]$	$[0,1]$	$[0,1]$	$[\![27,40]\!]$	$[0,1]$	$[0,1]$	$[\![0,40]\!]$			
$\mathcal{D}_{9,1}$	$[0,0.1]$	$[0.1,0.8]$	$[0,1]$	$[0,1]$	$[\![27,40]\!]$	$[0,1]$	$[0,1]$	$[\![0,40]\!]$	$[\![0,30]\!]$		
$\mathcal{D}_{9,2}$	$[0,1]$	$[0,1]$	$[0,1]$	$[0,1]$	$[\![27,40]\!]$	$[0,1]$	$[0,1]$	$[\![0,40]\!]$	$[\![0,30]\!]$		
$\mathcal{D}_{10,1}$	$[0,0.1]$	$[0.1,0.8]$	$[0,1]$	$[0,1]$	$[\![27,40]\!]$	$[0,1]$	$[0,1]$	$[\![0,40]\!]$	0	$[\![20,30]\!]$	$[\![0,60]\!]$
$\mathcal{D}_{10}$	$[0,1]$	$[0,1]$	$[0,1]$	$[0,1]$	$[\![0,40]\!]$	$[0,1]$	$[0,1]$	$[\![0,30]\!]$	0	$[\![0,30]\!]$	$[\![0,60]\!]$
$\mathcal{D}_{10}$	$[0,1]$	$[0,1]$	$[0,1]$	$[0,1]$	$[\![0,40]\!]$	$[0,1]$	$[0,1]$	$[\![0,30]\!]$	0	$[\![0,30]\!]$	$[\![0,60]\!]$

(b) Senescence entry assumed different for *type A* and *type B* cells

Supplementary Methods Table 3: Tables of the domains in which parameter estimations were run.

Weigh ω	Value before renormalization $(\omega_{sen}, \omega_{sen_A}, \omega_{sen_B}, \omega_{nta})$
ω^1	$(0.5, 0.9, 0.9, 1)$
ω^2	$(0.5, 0.5, 0.5, 1)$
ω^3	$(0.3, 1, 1, 1)$
ω^4	$(1, 1, 1, 1)$
ω^ℓ	$(148, 64, 84, 115)$

Supplementary Methods Table 4: Different values tested for the weight ω used in definition (5.2) of the cost function. It indicate how to weight the four errors between each of the (four) simulated and experimental G-graphs. Note that ω^ℓ corresponds to the number of generations composing each associated experimental graph ($\omega_i^\ell = n_i$, $i \in \{sen, sen_{_A}, sen_{_B}, nta\}$).

Weight ω	Population sizes N	Value of the cost		$r_{B\%}$	
		E_ω	E_{ω^3}	average	percentiles
ω^1	$(58, 64, 70)$	20.58	20.02	76.1%	$[69, 82]$
	(58)	21.36	21.02	80.0%	$[74, 86]$
ω^2	(58)	33.30	33.10	49.3%	$[36, 63]$
ω^3	$(58, 64, 70^*)$	16.54	16.54	52.5%	$[44, 61]$
	(58)	32.11	32.11	71.4%	$[64, 78]$
ω^4	$(58, 64, 70)$	21.09	19.75	76.6%	$[70, 83]$
	(58)	21.70	21.28	79.6%	$[73, 80]$
ω^ℓ	$(58, 64, 70)$	21.41	19.75	73.9%	$[67, 80]$
	(58)	21.46	19.61	77.4%	$[71, 84]$
	(58)	22.51	20.82	72.9%	$[66, 80]$
	(58^*)	21.66	19.42	76.5%	$[70, 83]$
	$(58, 64, 70)$	21.72	20.15	71.8%	$[64, 79]$
	$(58, 64, 70)$	34.45	33.37	55.5%	$[47, 64]$
	$(58, 64, 70)$	38.35	34.57	61.0%	$[53, 69]$
	(58)	41.65	39.35	39.9%	$[31, 47]$

Supplementary Methods Table 5: Sensitivity of CMA-ES minimization results to the weight ω (used in the definition of the cost function E_ω to minimize). Estimation is performed on $\mathcal{D}_{8,2}$, with parameters $\sigma_0 = 0.5$, $\bar{e}_{B\%} = 25\%$, $N = (58, 64, 70)$. The average and $5th$ and $95th$ percentile of the proportion $r_{B\%}$ of *type B* lineages (last two columns) were taken among $k = 1000$ simulations of $n_{sen} = 148$ lineages.

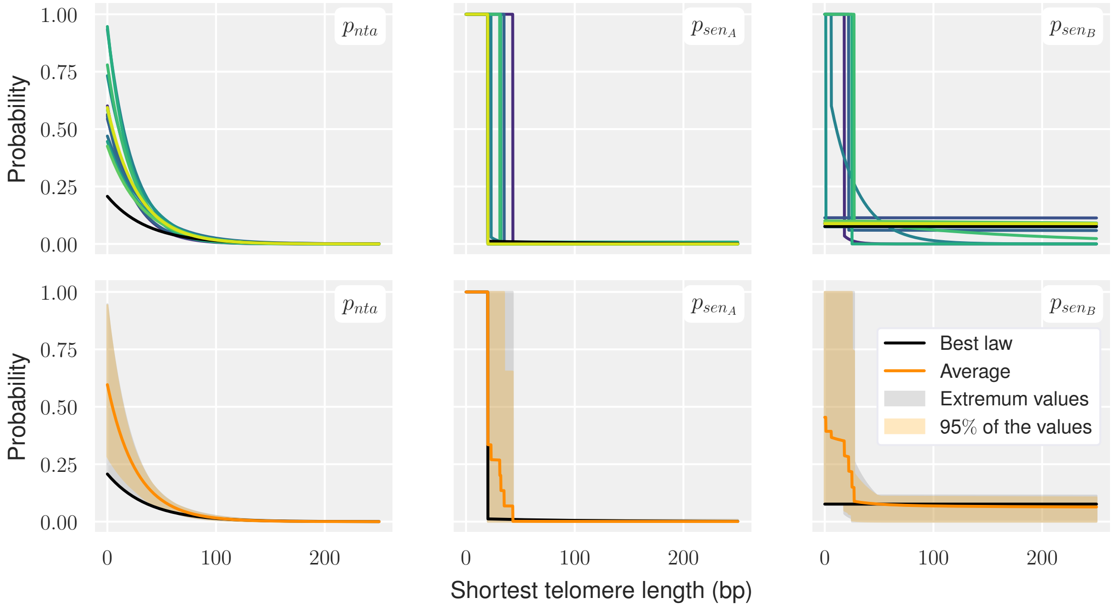

Supplementary Methods Fig. 13: Best fits of the laws $(\mathcal{P}_{nta})$, $(\mathcal{P}_{sen_A})$ and $(\mathcal{P}_{sen_B})$ retrieved when running several independent CMA-ES estimations for different values of ω (used in the definition of the cost function E_ω to minimize). Two different representations: *(top)* either plotting each fit individually, or the average fit with $5th$ and $95th$ percentile *(bottom)*. The black line *(bottom)* corresponds to the fit that best minimizes E_{ω^3}. The best fits of the laws are fairly close to each other, attesting that the estimation process is not very sensitive to the weight ω.

Same senescence law for *type A* and *type B* cells

The results are gathered in Supplementary Table 6 and Supplementary Methods Fig. 14.

Population sizes N	Minimum error		$r_{B\%}$	
	E_ω	E_{ω^3}	average	percentiles
$(70, 70, 70)$	16.94	19.11	25.91%	$[19, 33]$
$(70, 70, 70^*)$	17.21	19.17	26.6%	$[20, 34]$
$(70, 70, 70)$	17.26	19.17	24.4%	$[18, 32]$
$(70, 70, 70)$	27.72	28.01	65.5%	$[57, 73]$
$(70, 70, 70)$	28.32	28.12	43.0%	$[35, 51]$
$(70, 70, 70)$	28.32	28.12	43.0%	$[35, 51]$

Supplementary Methods Table 6: Sensitivity to weight $\mathcal{D} = \mathcal{D}_{5,1}$, $\omega = \omega^2$, $\sigma_0 = 0.5$, $\bar{e}_{B\%} = 40\%$.

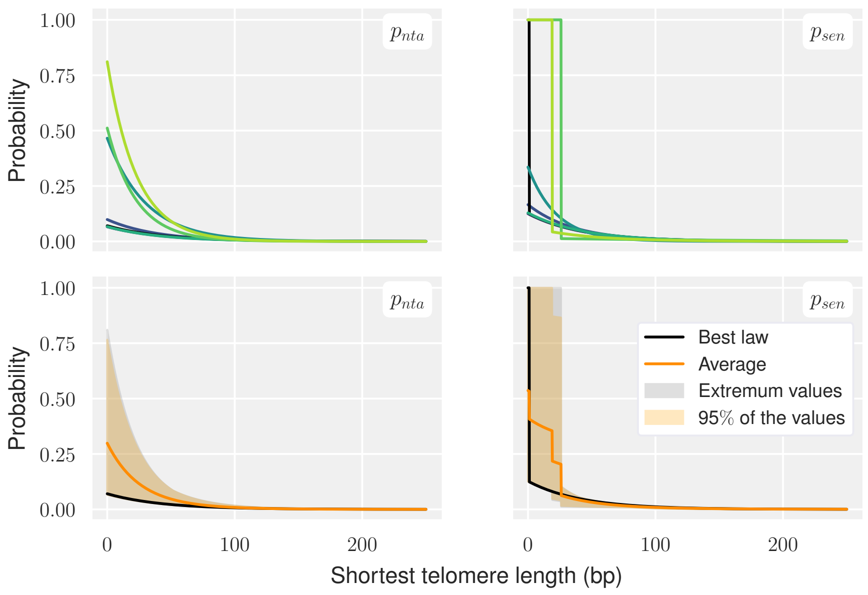

(a) $\mathcal{D} = \mathcal{D}_{5,1}$

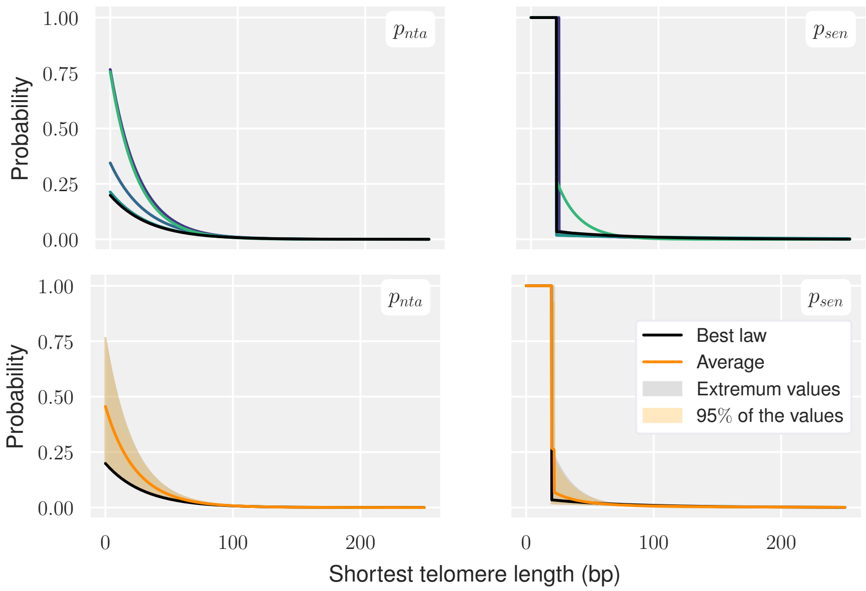

(b) $\mathcal{D} = \mathcal{D}_{5,2}$

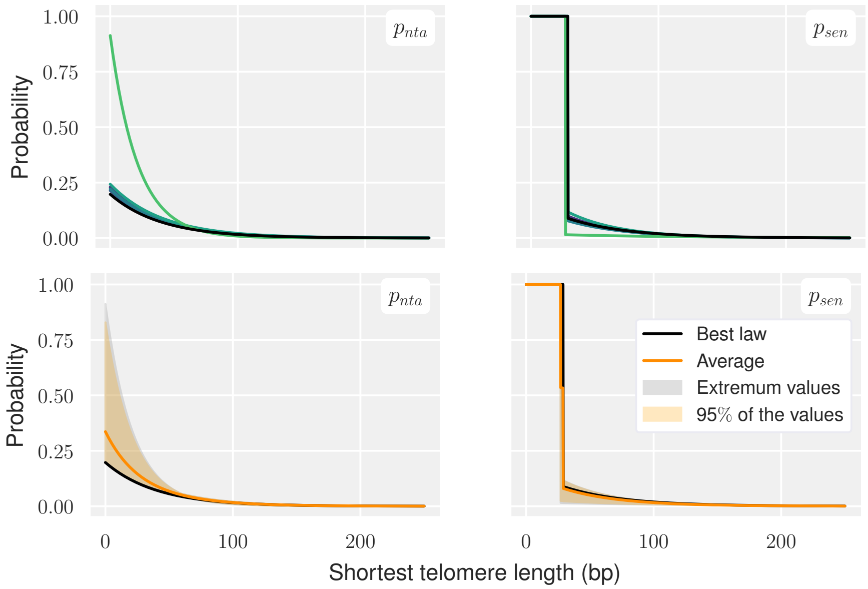

(c) $\mathcal{D} = \mathcal{D}_6$

Supplementary Methods Fig. 14: Best fits of the laws $(\mathcal{P}_{nta})$, $(\mathcal{P}_{sen})$ retrieved when running several independent CMA-ES estimations on different domains $\mathcal{D}$. Two different representations: *(top)* either plotting each fit individually, or the average fit with $5th$ and $95th$ percentile *(bottom)*. The black line *(bottom)* corresponds to the fit that best minimizes E_{ω^2}.

Different senescence law for *type A* and *type B* cells

Different types of modification of the initial distribution of telomere lengths were fitted in addition to the parameter of the laws $(\mathcal{P}_{nta})$ and $(\mathcal{P}_{sen_A})$ and $(\mathcal{P}_{sen_B})$.

Allowing translations of f_0. See Supplementary Methods Fig. 15.

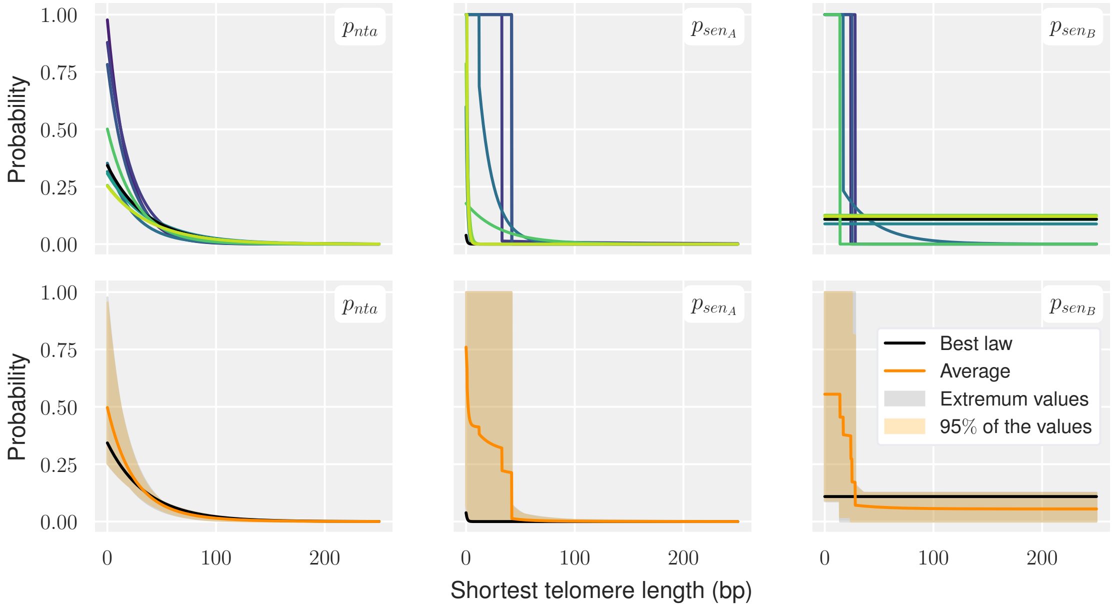

(a) $\mathcal{D} = \mathcal{D}_{8,1}$

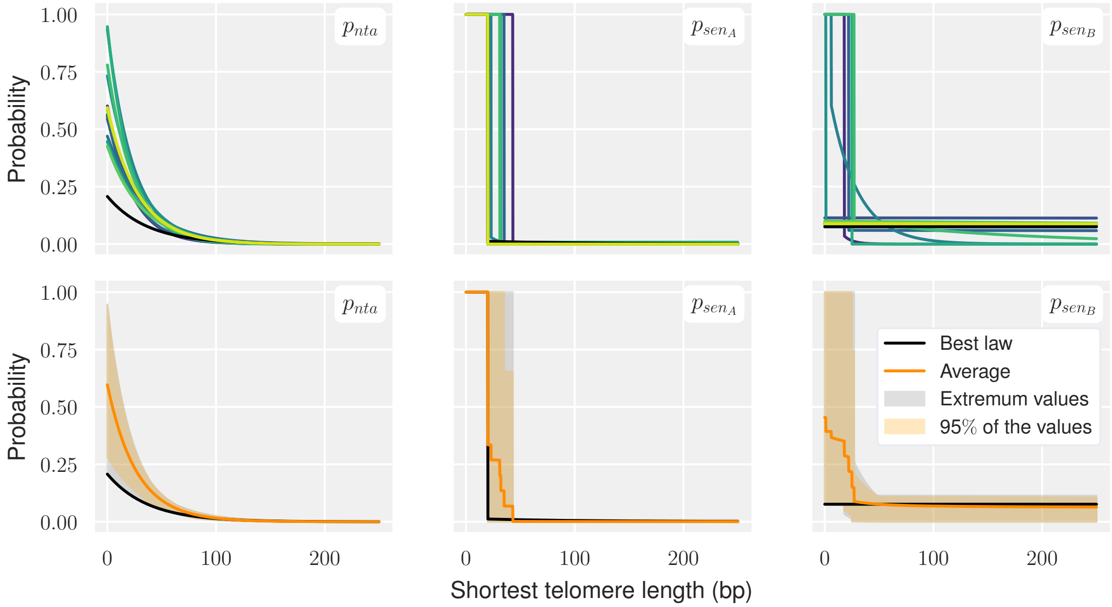

(b) $\mathcal{D} = \mathcal{D}_{8,2}$

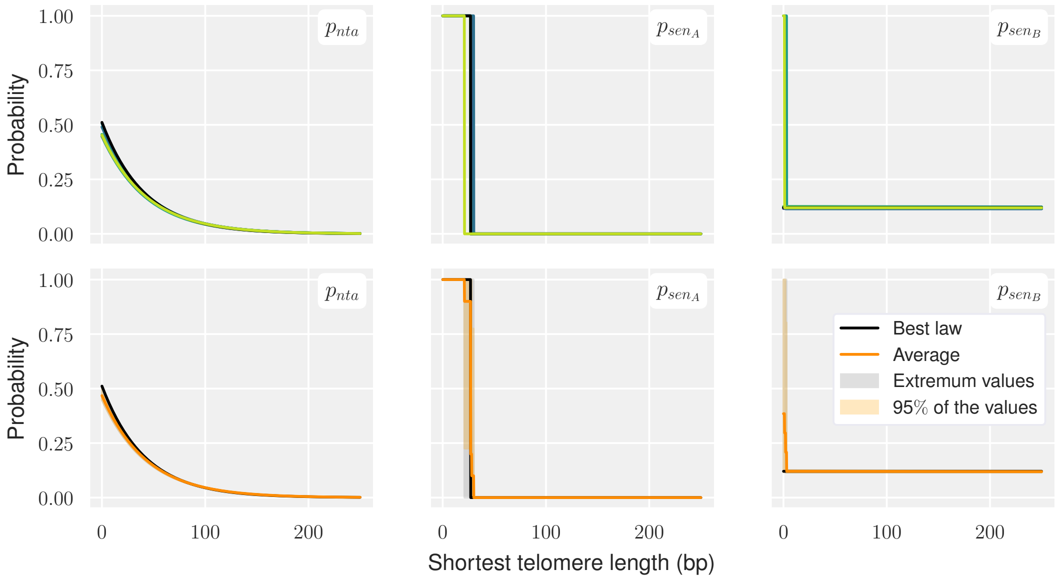

(c) $\mathcal{D} = \mathcal{D}_9$

Supplementary Methods Fig. 15: Best fits of the laws ($\mathcal{P}_{nta}$), ($\mathcal{P}_{sen_A}$) and ($\mathcal{P}_{sen_B}$) retrieved when running several independent CMA-ES estimations on different domains $\mathcal{D}$. Two different representations: *(top)* either plotting each fit individually, or the average fit with $5th$ and $95th$ percentile *(bottom)*. The black line *(bottom)* corresponds to the fit that best minimizes E_{ω^3}.

Allowing dilations of f_0. See Supplementary Methods Fig. 16.

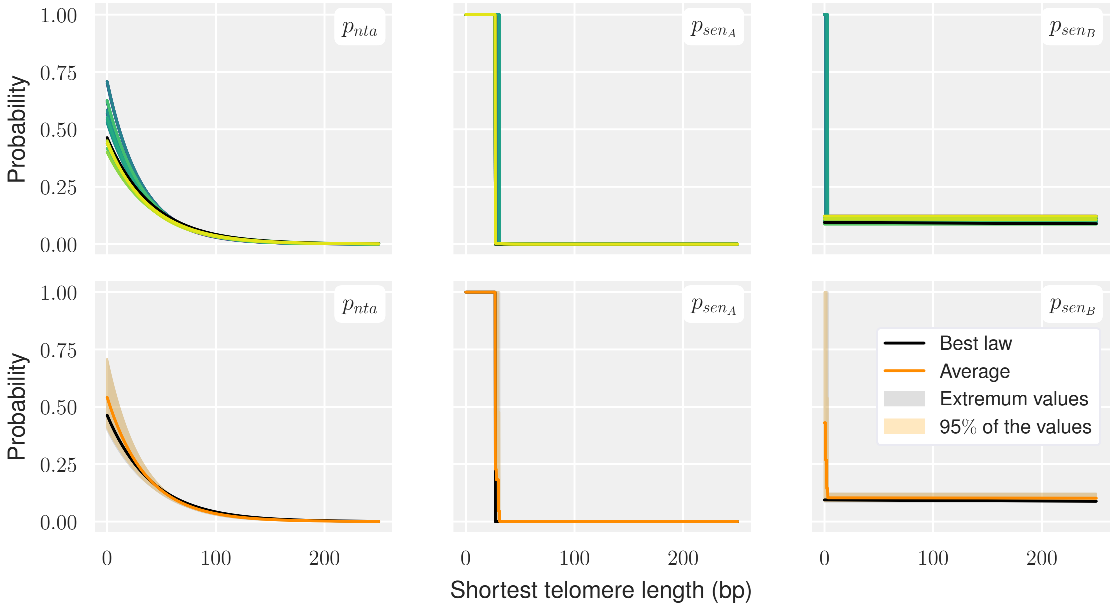

Supplementary Methods Fig. 16: Best fits of the laws $(\mathcal{P}_{nta})$, $(\mathcal{P}_{sen_A})$ and $(\mathcal{P}_{sen_B})$ retrieved when running several independent CMA-ES estimations on $\mathcal{D}_{10}$. Two different representations: *(top)* either plotting each fit individually, or the average fit with $5th$ and $95th$ percentile *(bottom)*. The black line *(bottom)* corresponds to the fit that best minimizes E_{ω^3}.

Supplementary References

1. Abdallah, P., Luciano, P., Runge, K. W., Lisby, M., Géli, V., Gilson, E. & Teixeira, M. T. A two-step model for senescence triggered by a single critically short telomere. *Nature Cell Biology* **11** (2009) (cit. on p. 6).
2. Auger, A. & Hansen, N. *A restart CMA evolution strategy with increasing population size* in *2005 IEEE Congress on Evolutionary Computation* **2.** ISSN: 1941-0026 (2005), 1769–1776 Vol. 2 (cit. on p. 15).
3. Bourgeron, T., Xu, Z., Doumic, M. & Teixeira, M. T. The asymmetry of telomere replication contributes to replicative senescence heterogeneity. *Scientific Reports* **5** (2015) (cit. on pp. 3, 13).
4. Martin, H., Doumic, M., Teixeira, M. T. & Xu, Z. Telomere shortening causes distinct cell division regimes during replicative senescence in Saccharomyces cerevisiae. *Cell & Bioscience* **11** (2021) (cit. on pp. 13–15).
5. Toure, C., Auger, A. & Hansen, N. Global linear convergence of evolution strategies with recombination on scaling-invariant functions. *Journal of Global Optimization* **86,** 163–203. ISSN: 1573-2916 (2023) (cit. on p. 17).
6. Xu, Z., Duc, K. D., Holcman, D. & Teixeira, M. T. The Length of the Shortest Telomere as the Major Determinant of the Onset of Replicative Senescence. *Genetics* **194** (2013) (cit. on pp. 3, 6).
7. Xu, Z., Fallet, E., Paoletti, C., Fehrmann, S., Charvin, G. & Teixeira, M. T. Two routes to senescence revealed by real-time analysis of telomerase-negative single lineages. *Nature Communications* **6** (2015) (cit. on p. 4).
